# The Ministry of Health

**Published:** 1920-12-18

**Authors:** 


### THE MINISTRY OF HEALTH.

The future fulfilment of charity's ideals and the nation's practical realisation of its duty towards the voluntary hospital system undoubtedly depends upon the coming of a new phase in the education of those classes in whose hands a sufficiency of money now lies. But we are also very certain that recent tendency on the part of a leading section of the daily press to evoke public criticism of the new Ministry is in every way harmful to this educative process. Therefore it is not out of place in this, our Special Appeal Number, to draw attention to certain points in hospital and political matters upon which a considerable number of people have been grossly misled.

To read article after article, the direct inference from which must be that the Minister of Health is scheming to place the hospitals upon a wholly rate-supported basis, and so to deprive them of their independence and voluntary character, is calculated to do harm far wider than the mere production of political opposition. The attitude of the ratepayer inevitably develops into a refusal to pay his share towards hospital maintenance. He comes to assume that later on he will have to *pay*, and sees no reason why he should *give* now.

But we can assure those to whom this appeal is addressed that the Government appears in no way opposed to the principle of obtaining and allowing for, now and in future, all possible voluntary assistance for the hospitals. Dr. Addison's reiterated statements in the House and elsewhere seem to have been deliberately unobserved and unmentioned by his political opponents. In the past twelve months both the Ministry and the hospitals themselves have been faced with financial problems of difficulty unexampled in the institutional world. Dr. Addison and some at least of the hospitals have already taken commendably energetic measures to deal with the situation, and we believe that throughout the premier consideration has been that the voluntary hospitals should not have to close their doors through lack of funds. The Government has on every occasion given encouragement to conferences and emergency meetings likely to aid in averting a catastrophe the magnitude of which the "hospital public," if not the general public, knows only too well. A way out of the difficulty must be found, and we should at least appreciate the fact that upon the Ministry now largely devolves the responsibility of devising a means—and co-ordinating voluntary and official endeavours towards that end. Despite the action of the House of Lords, legislation of some kind affecting hospitals must be made next year.

Emphatically it is not a case of war between officialdom and the voluntary principle. The progress of recent considerations given to the now famous Clause 11 of the Miscellaneous Provisions Bill clearly indicated the genuine necessity and will to co-ordinate as much as possible of the old system into the arrangements of the future and to preserve all that is best in the voluntary system. Description of their decision as a reprieve for the hospitals, represents the natural feeling of those who feared for a moment that financial difficulties were so hopelessly urgent that there was not time to achieve the ideal arrangement. But now another attempt is to be made, and, we believe, a well-directed one.

Let us remember that the Ministry's essential function is one of co-ordination, and that the amount of work it has before it is stupendous. Constructive criticism must at all times be helpful and to the public good, but the deliberate production of mistrust in the Ministry's work, in the voluntary hospital or any other connection, is at the present time quite unjustifiable. There is every indication that Dr. Addison is a strong advocate, personally and politically, of public support of the existing voluntary system. Let the people who can give see to it that such trust is not misplaced. Our appeal is made in the name of humanity, and there should indeed be no political brake to the wheels of its progress.

